# Effects of valproic acid on wound healing of the abdominal wall musculoaponeurotic layer: an experimental study in rats

**DOI:** 10.1590/0100-6991e-20243676-en

**Published:** 2024-05-24

**Authors:** RACHEL BIONDO SIMÕES, MARIA DE LOURDES PESSOLE BIONDO SIMÕES, SÉRGIO OSSAMU IOSHII, ROGÉRIO RIBEIRO ROBES, MOACIR OLIVEIRA DALL’ANTONIA, MATHEUS PRINCE GOEHR, PEDRO JUAN FURTADO NEVES

**Affiliations:** 1 - Universidade Federal do Paraná, Programa de Pós-graduação em Clínica Cirúrgica - Dep. de Cirurgia - Curitiba - PR - Brasil; 2 - Universidade Federal do Paraná, Técnica Cirúrgica e Cirurgia Experimental - Curitiba - PR - Brasil; 3 - Universidade Federal do Paraná, Departamento de Patologia da UFPR - Curitiba - PR - Brasil

**Keywords:** Valproic Acid, Epigenesis, Genetic, Wound healing, Abdominal Wall, Ácido Valproico, Epigênese Genética, Cicatrização, Parede Abdominal

## Abstract

**Introduction:**

valproic acid (VPA), an epigenetic drug, has potential for the treatment of neoplasms. Its effects on the healing of the peritoneal-musculo-aponeurotic plane (PMA) of the abdominal wall are studied.

**Method:**

sixty Wistar rats were allocated into two groups: experimental (VPA) and control (0.9% sodium chloride), treated daily, starting three days before the intervention and until euthanasia. Under anesthesia, a median laparotomy was performed and repaired with two synthetic layers. Assessments took place 3, 7 and 14 days after surgery. The integrity of the wounds, the quality of the inflammatory reaction, the intensity of the leukocyte infiltrate, collagen synthesis, the intensity of angiogenesis and the presence of myofibroblasts were studied.

**Results:**

there was dehiscence of the PMA plane in 11 of the 30 animals (p=0.001) in the experimental group. There was no difference in the quality and intensity of the inflammatory reaction. Immunohistochemistry revealed, in the experimental group, less collagen I (p3=0.003, p7=0.013 and p14=0.001) and more collagen III (p3=0.003, p7=0.013 and p14= 0.001). Collagen evaluated by Sirus Supra Red F3BA showed, in the experimental group, less collagen at all three times (p<0.001) with less collagen I and collagen III (p<0.001). A lower number of vessels was found on the 3rd day (p<0.001) and on the 7th day (p=0.001) and did not affect the number of myofibroblasts.

**Conclusion:**

VPA showed dehiscence of the PMA plane, with less deposition of total collagen and collagen I, less angiogenic activity, without interfering with the number of myofibroblasts.

## RESUMO

**Introdução:**

o ácido valpróico (VPA), droga epigenética, apresenta-se com potencial para o tratamento de neoplasias. Estudam-se seus efeitos sobre a cicatrização do plano peritônio-músculo-aponeurótico (PMA) da parede abdominal.

**Método:**

sessenta ratos Wistar, foram alocados em dois grupos: o experimental (VPA) e o controle (cloreto de sódio 0,9%), tratados diariamente, iniciando três dias antes da intervenção e até a eutanásia. Sob anestesia, fez-se uma laparotomia mediana que foi reparada com dois planos de síntese. As avaliações aconteceram 3, 7 e 14 dias após a cirurgia. Estudou-se a integridade das feridas, a qualidade da reação inflamatória, a intensidade do infiltrado de leucócitos, a síntese do colágeno, a intensidade da angiogênese e a presença de miofibroblastos.

**Resultados:**

o plano PMA mostrou-se deiscente em 11 dos 30 animais (p=0,001) do grupo experimento. Não houve diferença na qualidade da reação inflamatória e nem no infiltrado de leucócitos. A imuno-histoquímica revelou, no grupo experimento, menos colágeno I (p3=0,003, p7=0,013 e p14=0,001) e mais colágeno III (p3=0,003, p7=0,013 e p14= 0,001). Colágeno avaliado pelo Sirus Supra Red F3BA mostrou, no grupo experimento,menos colágeno nos três tempo (p<0,001) com menos colágeno I e colágeno III (p<0,001). Constatou-se menor número de vasos no 3º dia (p<0,001) e no 7º dia (p=0,001) e não afetou a quantidade de miofibroblastos.

**Conclusão:**

o VPA mostrou deiscências do plano PMA, com reação inflamatória semelhante.ao controle, menor deposição de colágeno total e de colágeno I, menor atividade angiogênica, sem interferir na quantidade de miofibroblastos.

## INTRODUCTION

Cancer, a health problem on a global scale, is the second leading cause of death in the world^1^, and alarming numbers are expected for the coming years^2^ ^,^ ^3^. This condition will imply high costs for the health system, the search for efficient treatments and prevention methods thus becoming imperative.

Neoplasms are characterized by anomalous and disordered cell growth and are dependent on genetic and epigenetic factors^5^ ^-^ ^7^. Molecular alterations provide tumors with evasion of growth suppressors, evasion of the immune system, permission of replicative immortality, pro-tumor inflammation, activation of metastasis and invasion, mutations and instability of the genome, resistance to tumor death, dysregulation of cellular energy metabolism, sustenance of proliferative signaling, and induction of angiogenesis^8^. 

Epigenetics is an area of biology that involves alterations in gene expression without modifications in the DNA sequence. The main mechanisms involved are related to DNA methylation and histone alterations, especially deacetylation^9^. DNA methylation involves the enzymes DNA methyltransferases, which add the methyl radical at position 5 of the cytokine ring (C5), alterations that affect mainly the sites known as CpG islands, which are regions of the DNA chain rich in CpG dinucleotides. Alterations in the methylation of these regions can trigger the inhibition of tumor suppressor genes or promote the activation of oncogenes, promoting the development of cancer^10^. Methyltransferase and histone deacetylase inhibitors have emerged as therapeutic alternatives in some types of cancer^6^.

Several mechanisms are associated with the alteration of histone chains. The acetylation of lysine residues present in these histones, mediated by the enzymes histone acetyltransferases (HAT) and histone deacetylases (HDAC), is related to the regulation of gene transcription, apoptosis, autophagy, and cell cycle control^11^. Several drugs act on these epigenetic effects, prolonging patients’ survival^11^ ^-^ ^22^. Valproic acid (VPA) is one of these drugs, with the ability to inhibit histone deacetylases (HDACs) of classes I (HDACs 1, 2, 3, and 8) and II (HDACs 4, 5, 7, and 9)^23^ ^-^ ^28^ ^,^ ^29^. 

VPA has been used for decades for the treatment of bipolar disorders and epilepsy^29^. Recent articles report its ability to shrink some types of tumors and their invasive potential. It is considered an epigenetic drug because it is capable of leading to changes in the transcription, translation, and replication of genes, acting on the methylation of histone and non-histone proteins, with remodeling of chromatin and gene expression of non-coding RNAs (ncRNA)^29^. 

In breast cancer, VPA altered the proliferation, survival, cell migration, and expression of hormone receptors in tumor cells in preclinical and clinical settings^27^, and in bladder tumor cell lines, it decreased cell proliferation. With hypermethylated DNA, the G1 phase is blocked^30^ ^,^ ^31^. 

VPA has been found to have anti-angiogenic effects both in vitro and in vivo^27^ ^,^ ^32^, probably by downregulating pro-angiogenic genes, such as vascular endothelial growth factor (VEGF) and/or endothelial nitric oxide synthase (eNOS). In addition, the inhibition of HDACs leads to hyperacetylation of hypoxia-inducing factor 1-alpha (HIF-1α), a pro-angiogenic transcription factor, which is degraded^7^. 

Vessel neoformation is essential for healing^33^, and growth factor beta (TGF-β), basic fibroblastic growth factor (bFGF), and vascular endothelial growth factor (VEGF) are necessary for the reconstitution of injured tissues to occur^34^ ^,^ ^35^. Therefore, if on the one hand, angiogenesis deficiency could beneficially interfere with tumor growth, on the other, it could lead to inadequate healing^36^.

Alterations in healing could result not only from the inhibition of HDAC, with decreased cell proliferation and migration^27^ ^,^ ^31^ ^,^ ^37^, but also from thrombocytopenia^38^ ^-^ ^40^ and decreased levels of fibrinogen and VEGF^41^.

How would VPA influence healing? Some authors have reported hampered healing^40^ ^-^ ^42^, while others have concluded that there are beneficial effects^43^ ^-^ ^46^. 

In recent work of our line of research on cutaneous healing, we observed that the administration of VPA promoted a more intense inflammatory reaction and decreased angiogenesis and collagen deposition, especially type-I collagen. In another study by our group on healing of the urinary bladder, we noticed that VPA determined changes in the healing process, but no fistulas or dehiscence^48^. 

In the literature reviewed, we found no studies on the effects of VPA on aponeurotic healing. If we consider that this plane is formed by connective tissue that is dense, ordered, rich in collagen, but poor in vascularization, the use of VPA, especially in cancer patients, would generate concern when they need to undergo surgical interventions, especially accesses to the abdominal cavity made through the midline.

Thus, the objective of this study was to recognize, in a murine model, the effects of VPA on the healing process of the abdominal aponeurosis through the analysis of the inflammatory reaction, angiogenesis, and collagen synthesis. 

## METHODS

This study was evaluated and approved by the Ethics Committee on the Use of Animals of the Biological Sciences Sector (CEUA-Biológicas) of the Federal University of Paraná (UFPR) on June 18, 2019, under number 1313, proceeding 23075.058610/2019-42. It complied with Federal Law No. 11,794 of October 8, 2008 and followed the guidelines of the Brazilian Society of Science in Laboratory Animals (SBCAL/COBEA). To determine the sample size, we used data from previous experiments in this line of research for an alpha error of up to 0.05 (5%), sampling power of 1 - beta error. The purpose of this calculation was to respect the “3 Rs”.

We used 60 120-day-old male Rattus norvegicus albinus, Rodentia mammalia, of the Wistar lineage, provided by the Central Vivarium of the Federal University of Paraná. They weighed, on average, 462.33 ± 33.72g. They were randomly selected for the control (C) and experimental (E) groups and again separated into three subgroups of ten animals, according to the measurement moment, named C3, C7, C14, E3, E7, and E14.

The animals were kept in the Laboratory of the Discipline of Surgical Technique and Experimental Surgery of the Federal University of Paraná, with controlled relative humidity and temperature (20 ± 2º C), in a 12-hour light/dark cycle. They were given commercial chow suitable for the species and water ad libitum. 

The rats in the experimental group were administered 100 mg/kg/day of VPA^49^ by gavage. The medication was started three days before the intervention and continued until the day of euthanasia. The rats in the control group received an equivalent volume of 0.9% sodium chloride solution.

For the intervention, they were anesthetized using an intramuscular injection of 0.1 ml/100g of body weight of a solution consisting of 1.0 ml of ketamine (50 mg/ml) and 1.0 ml of xylazine (20 mg/ml). In addition, an intraperitoneal injection of 20mg/kg of thionembutal was performed. For anesthetic maintenance, isofluoraene 1%-1.5% associated with 100% oxygen was used under an inhalation mask.

The ventral abdominal wall was shaved, followed by fixation to the surgical clipboard and antisepsis with 2% chlorhexidine alcohol solution. 

We performed a median laparotomy of approximately five centimeters, followed by laparorhaphy with two synthesis planes: the musculoaponeurotic plane with continuous suture of a 5.0, multifilament, braided polyglactin 910 suture, and the skin plane with a 4.0 nylon monofilament continuous suture. All procedures were performed by a single surgeon.

After recovering from anesthesia and analgesia (tramadol hydrochloride, 5mg/kg, intramuscularly), the animals were returned to their cages and medicated daily until the day scheduled for measurement. The euthanasia took place after three, seven, and 14 days, performed by a veterinarian, under the guidelines of the euthanasia practice of CONCEA, Resolution No. 37 of the Ministry of Science, Technology, Innovation, and Communication (Brasília/DF) of February 22, 2018, which consists of anesthetic overdose by intraperitoneal injection. 

The wound was inspected for dehiscence and secretions. We resected a six-centimeter wall segment containing the surgical wound from all animals and fixed it in 10% formalin for histopathological study.

Four-micrometer-thick sections were made and stained with hematoxylin-eosin. The order of the scar and the intensity and quality of the inflammatory reaction were evaluated using the methodology of Vizzotto Junior et al.^50^. The parameters were classified as pronounced, moderate, discrete, and absent, and transformed into quantitative variables. The indices that characterized acute inflammatory process were assigned a negative sign, and the chronic inflammatory index was assigned a positive sign, ranging from -3 to +3. The summation provided a final score that allowed the classification of the inflammatory process as acute, sub-acute, and chronic^50^.

Sections stained using the picrosirius-red F3BA technique allowed collagen recognition^51^ ^,^ ^52^. The images were captured with a Sony CCD101^®^ camera and analyzed using MediaCybernetics’ Image-Plus^®^ 4.5 for Windows. Ten fields with 400-fold magnification were analyzed, each field with an area of 142.901 μm^2^. In each field, the percentage occupied by collagens I and III was determined. Since the other types of collagen represent very small fractions, the sum of collagens I and III was considered to be the total collagen of the scar. The average of the ten fields read was obtained and considered for each animal.

New sections were submitted to five different immunomarkers using the streptavidin-biotin-peroxidase technique, as described by Hsu, Raine, and Fanger^53^. The common anti-leukocyte monoclonal antibody (anti-ACL) was used for the identification and quantification of leukocytes, anti-CD34 was used for the identification of endothelial cells and vessels, anti-collagen I and anti-collagen III antibodies were used to recognize collagens, and anti-alpha-smooth muscle (anti-AMS) was used to identify myofibroblasts. The images were scanned with the Axio Scan.Z1 Digital Slide Scanner^®^ (Zeiss, Germany) and validated by the Zeiss ZenLite^®^ software (Zeiss, Germany). For these evaluations, 10 fields of 131,307.264 μm^2^ were read. 

The results of quantitative variables were described as mean, standard deviation, median, minimum, maximum, and interquartile range (IQR). Categorical variables were described by frequency and percentage. To compare quantitative variables between the Control and Experiment groups, we used the Student’s t-test for independent samples or the non-parametric Mann-Whitney test. The comparison of the days of euthanasia (three, seven, and 14) was made using the one-way analysis of variance (ANOVA) model and the Bonferroni’s post-hoc test or the non-parametric Kruskal-Wallis test and the Dunn’s post-hoc test. The Fisher’s exact test was used for the analysis of categorical variables. Values of p<0.05 indicated statistical significance. For multiple comparisons of the days of sacrifice, the p-values were corrected by Bonferroni. Data were analyzed using IBM SPSS Statistics v.28.0. Armonk, NY: IBM Corp.

## RESULTS

**Figure 1:**
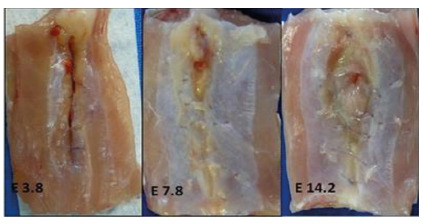
Aspects observed on the abdominal walls of the animals in the experiment group that presented dehiscences of the musculoaponeurotic layer.

No secretion was found in the wounds at any of the observed times. The skin showed similar signs of epithelialization in the three moments evaluated. However, the aponeurotic plane was dehiscent in 11 of the 30 animals in the experimental group, representing 35.67% of the sample (p=0.0001), one on the third day (10%), four on the seventh day (40%), and six on the 14th day (60%). As time went by, the number of animals with dehiscence increased (Figure 1).

**Figure 2:**
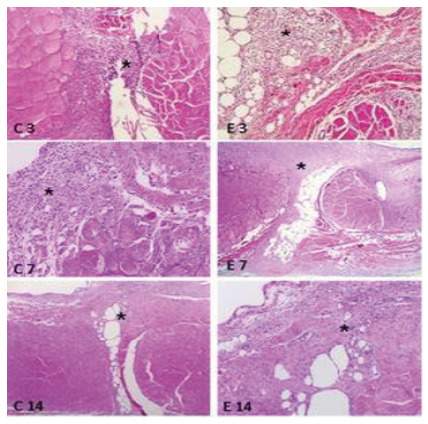
Photomicrographs of histological sections of the musculoaponeurotic layer showing aspects of inflammatory reaction (HE-200X).

The inflammatory reaction on the third day was acute or sub-acute in both groups. At seven days, the inflammatory reaction was acute or sub-acute in all histological sections of the experimental group, while in the control group, in four of the ten there was a chronic reaction (p=0.087). At 14 days, in the histological sections of the wounds in the control group the chronic condition predominated, while in the experimental group the sub-acute condition predominated (p=0.179). (Table 1, Figure 2). 

**Table 1:** Quality of the inflammatory reaction.

Inflammatory reaction	3 days		7 days		14 days	
	Control	Experiment	Control	Experiment	Control	Experiment
Acute/sub-acute	11	10	6	10	3	7
	100%	100%	60%	100%	30%	70%
Chronic	0	0	4	0	7	3
	0%	0%	40%	0%	70%	30%
Total	11	10	10	10	10	10
p* (Contr. Exper.)	1		0,087		0,179	

*Fisher’s exact test p<0,05.

The number of leukocytes marked by anti-ACL was higher in the control group on the third day, but the difference was not significant (p=0.197). On the seventh and 14^th^ days, the count of these cells was higher in the experimental group, but without significant difference (seven days p=0.165 and fourteen days p=0.315) (Table 2).

**Table 2:** Average WBC in 10 fields (marked by anti-LCA).

	Day	Group	n	Average	Standard deviation	Median	Minimum	Maximum	AIQ	p* Contr. x Exper.
	3	Contr.	11	47,27	23,70	40	25	100	30	
		Exper.	10	38,40	19,24	30	24	80	20	0,197
	7	Contr.	10	19,00	9,94	15	10	35	20	
		Exper.	10	33,50	20,28	30	5	65	30	0,165
	14	Contr.	10	7,90	2,18	8	5	10	5	
		Exper.	10	10,60	5,32	10	5	20	2	0,315
				D3xD7xD14	D3 x D7	D3 x D14	D7 x D14			
p**		Contr.		:	:	:	:			
				p=<0,001	p=0,050	p<0,001	p=0,058			
				D3xD7xD14	D3 x D7	D3 x D14	D7 x D14			
		Exper.		:	:	:	:			
				p<0,001	p=1	p=0,001	p=0,008			

*Non-parametric Mann Whitney test (LCA) or Student test for independent samples (p<0,05). **Non-parametric Kruskal-Wallis test (LCA) (p<0,05). AIQ: interquantum amplitude.

**Table 3:** Average number of blood vessels in 10 fields (anti-CD34).

	Day	Group	n	Average	Standard deviation	Median	Minimum	Maximum	AIQ	p* Contr. x Exper.
	3	Contr.	11	17	1,9	18	14	20	3	
		Exper.	10	11,6	2,12	12	8	14	2	<0,001
	7	Contr.	10	18,1	2,6	18	15	22	5	
		Exper.	10	13,1	3,18	14	6	17	3	0,001
	14	Contr.	10	10	2,67	9,5	7	15	3	
		Exper.	10	8,2	2,53	8	5	12	4	0,139
				D3xD7xD14	D3 x D7	D3 x D14	D7 x D14			
p**		Contr.		:	:	:	:			
				p<0,001	p=0,908	p<0,001	p<0,001			
				D3xD7xD14	D3 x D7	D3 x D14	D7 x D14			
		Exper.		:	:	:	:			
				p=0,001	p=0,647	p=0,023	p<0,001			

*Teste t de Student para amostras independents: p<0,05. **Teste ANOVA com um fator (CD34): p<0,05. AIQ: amplitude interquântica.

The mean number of vessels was significantly lower in the experimental group at three days (p<0.001) and seven days (p=0.001). In the 14-day analysis, although the experimental group showed a smaller number of vessels, the difference was not significant (p=0.139) (Table 3, Figure 4).

**Figure 3:**
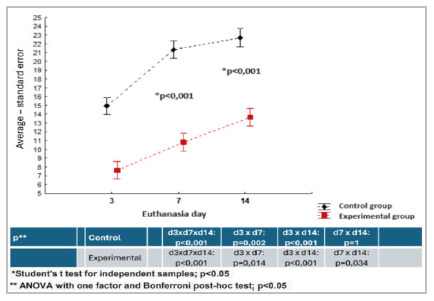
Area occupied by collagen, evaluated by Picrosirius-red F3BA.

**Figure 4:**
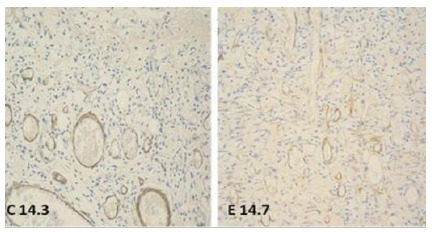
Photomicrographs demonstrating the presence of blood vessels, on day 14 (anti-CD34, 200 X).

Myofibroblasts were rare in both groups on the third day (p=0.361). They existed in moderate quantity on the seventh day (p=1) and on the 14th day (p=0.087).

In the three moments, the analysis of collagen by immunohistochemistry showed a higher amount of collagen I in the control group and a higher amount of collagen III in the experimental group (three days p=0.003, seven days p=0.013, and 14 days p=0.001).

**Figure 5:**
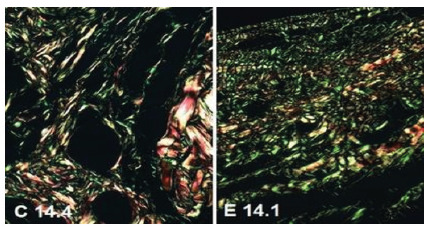
Photomicrographs of histological sections on day 14, stained by Picrosirius-red F3BA (400 x) (green = type III collagen, yellow to red = type I collagen).

The analysis of collagen with picrosirius-red F3BA enabled evaluation of the areas and computing the averages, showing a lower amount of total collagen in the experimental group in the three moments evaluated (p<0.001). However, there was a gain in both groups as the process progressed (Figure 3). Figure 5 shows the aspects of collagen.

After analyzing the concentrations of collagen fractions, more type I and type III collagen were found in the control group in the three moments (p<0.001).

## DISCUSSION

Wound healing in all tissues behaves similarly. The differences are evident in the time of evolution, which is related to the replicative capacity of the cells and the conditions of the environment. It is a complex process that involves numerous different cells, and is didactically divided into inflammatory, proliferative, and remodeling phases^36^ ^,^ ^54^ ^,^ ^55^. 

There is a lot of information on the histological evolution of the healing process, but much remains to be discovered about its molecular basis. Epigenetic regulation, including histone modification and DNA methylation, probably participates in this process^55^.

Although there are several mechanisms of histone modification, acetylation and methylation are the most understood. While methylation participates in gene activation and repression, acetylation is associated with gene activation^57^. 

Macrophages are essential cells in the healing process, participating in all phases, with phenotypes that transition from pro-inflammatory to anti-inflammatory. Apparently, this modification is regulated by histone methyltransferases^55^. 

The granulation tissue initially has more collagen III. Collagen I, being a more complex molecule, is added to the extracellular matrix more slowly. Fibroblasts are responsible for the synthesis of collagen, but also for the synthesis of fibronectin, elastin, laminins, proteoglycans, hyaluronic acid, and glycoproteins^58^ ^,^ ^59^.

Although HDAC inhibitors have been shown to cause accumulation of acetylated histones, inhibiting the growth of keratinocytes, they do not seem to inhibit fibroblasts^60^.

Depending on which histone is being deacetylated, different effects are observed. Deacetylases remove acetyl groups from histones leading to chromatin condensation, preventing transcription, and thus blocking cell replication^27^. By removing the acetyl radical, HDAC2, a class-I histone, inhibits the expression of growth factors. Among these factors are insulin growth factor I (IGF-I), fibroblast growth factor 10 (FGF-10), and epidermal growth factor (EGF)^61^.

It was experimentally demonstrated that the topical application of VPA in wounds reduced the area and promoted greater migration of keratinocytes. The authors also reported that they found suppression of apoptosis and a higher concentration of collagen. Thus, at the cutaneous level, there would be an improvement in healing^43^. In another experiment using intraperitoneal administration, the results were similar^44^. However, in a study of our line of research on cutaneous healing, we noticed that the administration of VPA promoted a decrease in angiogenesis and collagen deposition, especially type-I collagen^47^. 

An experiment in pigs demonstrated that VPA increases fibrinolysis^45^. Levels of α-SMA, which identify contractile proteins and therefore myobifroblasts, as well as markers for collagens I and III, were increased in wounds treated by VPA in another study^46^.

In this study, the inflammatory reaction showed a very similar evolution in both groups qualitatively. The analysis of inflammatory cellularity evaluated by the anti-ACL marker allowed us to verify the intensity of these cells’ infiltrate. We found more leukocytes in the histological sections of the control group on the third day and more in the other two moments in the group treated with VPA. However, this difference was not significant at any moment. This leads us to hypothesize that there may have been a delay in the development of the inflammatory phase, but that there was no significant influence on the inflammatory process. 

The experiments reported in the reviewed articles show an increase in collagen I and III when VPA was used, but on skin wounds. In this experiment, when using immunohistochemistry staining, we observed less collagen I in the sections obtained from the specimens of the treated animals and more type-III collagen. The confirmation was made by the analysis under polarized light of the sections stained by picrosirius-red F3BA. We found more collagen in the aponeurosis scars of the controls, with a higher percentage of both type-I and type-III collagens. It is noteworthy that quantification by the picrosirius-red F3BA method allows a better analysis of the areas. Even so, the higher concentration of total collagen and the collagen I fraction in the control group allows us to suppose that the fibroplasia process may be compromised in the group treated with VPA.

Angiogenesis is essential for tissue maintenance and homeostasis. In cancer biology, the network of vessels is crucial for the supply of nutrients and oxygen to the tumor, and new blood circuits are essential for the maintenance of tumor cells^62^. The regulatory mechanisms in physiological angiogenesis are coordinated, well-balanced, and strictly regulated by pro- and anti-angiogenic factors. In contrast, tumor angiogenesis is characterized by an excess of pro-angiogenic factors that lead to the proliferation of uncoordinated endothelial cells (EC) and the migration of support cells^63^. It is worth remembering that anti-angiogenic effects have been attributed to VPA. In vitro, it inhibited endothelial cell proliferation, reduced vascular endothelial growth factor (VEGF) secretion, and inhibited tube formation in the angiogenesis assay^32^. This effect is credited to the downregulation of pro-angiogenic genes and histone deacetylase inhibitors (HDAC) that would lead to hyperacetylation of HIF-1α, which is a pro-angiogenic transcription factor^7^. 

VPA, therefore, has anti-angiogenic potential and would be useful in the treatment of cancer. It has also been found to be involved in the reduction of endothelial nitric synthetase (eNOS) expression preceded by HDAC inhibition^42^, and a decrease in NO levels has been observed, which participates in endothelial proliferation, migration, and angiogenesis organization^43^ ^,^ ^64^ ^-^ ^66^. 

In this study, we observed that the group treated with VPA developed a lower number of vessels. Apparently, VPA decreases cyclic guanylate-monophosphate and eNOS, so VEGF would remain low. As a result, there would be a lower rate of endothelial cell replication and less migration and organization of vessels^7^ ^,^ ^27^ ^,^ ^32^ ^,^ ^42^ ^,^ ^66^.

However, the neoformation of vessels is essential for healing^33^. Thus, if on the one hand, angiogenesis deficiency can be beneficial, reducing tumor growth, on the other, it could lead to inadequate healing^36^.

The smaller number of vessels found in this study may partially explain the lower deposition of collagen, since this synthesis is oxygen-dependent.

Although we found changes in the intensity of the inflammatory reaction, which constitutes the initial phase, as well as lower collagen density and a lower number of neovessels, there was no difference in the number of myofibroblasts.

The smaller number of vessels and the lower amount of collagen may be the reason for finding dehiscence of the aponeurotic plane in more than one third of the sample (p=0.0001). It should be noted that this condition gradually increased over time, reaching 60% of the animals on the 14^th^ day. In studies carried out by this group, we found that in skin and urinary bladder healing, these reductions were present, but did not cause macroscopic damage. At the aponeurotic level, however, there was significant damage, perhaps due poorer circulation and the need for a greater amount of collagen to perform resistance, and this operation was oxygen-dependent.

It is important to recognize these alterations so that when intervening in a cancer patient who has received or is receiving treatment associated with epigenetics, specially the VPA, extra care is taken, because in addition to the damage to healing resulting from chemotherapy or even radiotherapy, there are also those caused by epigenetic blockages.

## CONCLUSION

Administration of VPA led to decreased angiogenesis and deposition of collagen, especially type-I collagen, with dehiscence of the aponeurotic plane.
